# The effectiveness of Dental Health Support Workers at linking families with primary care dental practices: a population-wide data linkage cohort study

**DOI:** 10.1186/s12903-018-0650-z

**Published:** 2018-11-21

**Authors:** Faith Hodgins, Andrea Sherriff, Wendy Gnich, Alastair J. Ross, Lorna M. D. Macpherson

**Affiliations:** 1 School of Philosophy, Psychology and Language Sciences, University of Edinburgh, 7 George Square, Edinburgh, UK; 2 Glasgow Dental School, School of Medicine, Dentistry and Nursing, College of MVLS, University of Glasgow, 378 Sauchiehall Street, Glasgow, UK

**Keywords:** Childsmile, Oral health, Dental public health, Health inequality, Lay health worker, Community health worker, Support worker, dental practice

## Abstract

**Background:**

Link workers (lay health workers, health support workers) based in the community provide additional support to individuals and families to facilitate engagement with primary care and other services and resources. This additional support aims to tackle the wider socio-economic determinants of health that lead to inequalities. To date, there is no clear evidence of the effectiveness of these programmes. This study evaluates the effectiveness of Dental Health Support Workers (DHSW) at linking targeted families with young children to primary care dental practices. The DHSW role is one component of Childsmile, the national oral health improvement programme in Scotland.

**Methods:**

A quasi-experimental approach captured the natural variation in the rollout of the DHSW intervention across Scotland in a cohort of children born between 2010 and 2013. Survival analysis explored “time to attendance” at primary care dental practice. Cox’s regression models compared attendance rates and time until first attendance between those families who received support from the DHSW and those who did not.

**Results:**

The cohort consisted of 35236 children. Thirty-three percent of the cohort (*n* = 11495) were considered to require additional support from a DHSW. Of these, 44% (5087) received that support. These families were more likely to attend a dental practice (Hazard Ratio [95% Confidence Interval] =1.87 [1.8 to 1.9]) and, on average, did so 9 months earlier (median time until first attendance: 8.8 months versus 17.8 months), compared to families not receiving additional support.

**Conclusions:**

Link workers (DHSW) within the Childsmile programme are effective at linking targeted children to primary care dental services and, most notably, *at a younger age* for prevention. This is the first study of its kind to evaluate the effectiveness of link-worker programmes using a robust quasi-experimental design on three, population-wide, linked datasets. These results will inform future health programmes which aim to improve health and reduce inequalities by reaching and supporting families from more disadvantaged backgrounds.

## Introduction

Link workers (lay health workers, health support workers) based in the community provide additional support to individuals and families to facilitate engagement with primary care and other services and resources [1]. This additional support aims to tackle the wider socio-economic determinants of health that lead to inequalities. Evidence of the effectiveness of these programmes is lacking and there is a need for robust evaluation of current programmes [2–4].

In response to decades of poor child oral health and widening inequalities in children in Scotland, “An action plan for improving oral health and modernising NHS dental services in Scotland” was published in 2005 [5]. As a response to the action plan, Childsmile, the national oral health improvement programme for children, was established [6]. The programme includes universal and targeted components to improve oral health and reduce inequalities. These components include: (1) nursery and school-based tooth brushing and fluoride varnish programmes; (2) targeted home-based support delivered by Dental Health Support Workers (DHSW) to promote good oral health within the community and to link children to primary care dental practice; and, (3) ongoing prevention delivered in primary care dental practices (including: fluoride varnish; oral hygiene demonstration; and, dietary advice).

In component (2) above, the DHSWs work with health visiting teams, and have families referred to them by health visitors, based on need. This is a targeted part of the Childsmile programme, aiming to address inequalities by providing additional support to families in proportion to their need. The role of the DHSW is to link these families with a dental practice, promote oral health behaviour change, and link families to wider community resources.

Childsmile is not unique in Scotland in its use of link workers. Recently, the Scottish government has funded the Links Worker Programme for areas of high deprivation across the country. The Community Links Practitioners all have backgrounds in community development or the third sector and work directly with General Medical Practices to identify individuals with complex social circumstances who may need additional support to access services and other community assets. The aim is to “mitigate the impact of the social determinants of health” to help people live well [7]. With two major public health interventions utilizing community-based link workers taking place in Scotland, it is timely to evaluate the effectiveness of the DHSW at linking families with a primary care dentist within the Childsmile programme.

This study aimed to link administrative health data from three national datasets to evaluate the effectiveness of the DHSW intervention at linking families with primary care dental services with respect to attendance and age at first attendance. A secondary aim is to assess the extent to which the intervention has reached the target population (i.e. those who require additional support to engage with dental services). We hypothesised that, among children deemed by health visitors to require DHSW support, the rates of dental attendance would be higher and age at first attendance lower for those who received the intervention.

## Materials and methods

### The DHSW intervention: Role, rollout and delivery

All newborn children in Scotland are visited by a health visitor (or public health nurse) around 6–8 weeks of age as part of the Early Years Referral Pathway (EYRP), which is the assessment and communication pathway for health visiting teams and other health services. As part of this visit, health visitors determine whether a family requires additional support with oral health by using their professional judgement to assess the child’s risk of poor oral health (e.g. whether child is breastfed or bottle fed, the family’s oral health habits at home, and the parents’ and siblings’ oral health and dental practice attendance). Health visitors refer families to a Dental Health Support Worker (DHSW) for this additional (mostly home-based) support. The role of the DHSW is to facilitate attendance of the family at a dental practice; to support oral health behaviour change and to link the family with other community initiatives. Once a family is referred by a health visitor, a DHSW will make contact when the child is around 3 months of age. The content of the DHSW intervention should be tailored to the family’s needs.

The intervention delivered by DHSWs was piloted in the West of Scotland health boards from 2006 onwards, with a rollout across every health board in the country from 2010. The intervention delivery is supported by an online data collection system for monitoring and evaluation purposes. In the early stages of rollout, some health boards had not yet implemented the referral pathway. For others, the referral pathway and local working practices were still undergoing refinement, often restricting the capacity to deliver the intervention. It also took more time in some areas for DHSW posts to be filled, although health visitors had started to make referrals. This natural variation in the delivery of the intervention is common in large public health programmes, especially in the early stages of implementation and provides a unique opportunity to robustly evaluate the intervention within the group of children targeted for the intervention.

### Study design

A quasi-experimental approach, which captured the natural rollout of the DHSW intervention across the country (as described above), was taken. We considered attendance at a primary care dental practice as the primary outcome.

Secondary health and administrative data from three national datasets were linked to form a birth cohort (*n* = 114097). Of these, the referral pathway was (at least partially) operational for *n* = 35236 (31%) children, who formed the cohort for this study.

Three groups were compared:

1. families who were referred to the DHSW by a health visitor and had an intervention delivered (Group 1);
2. families who were referred to the DHSW by a health visitor and did not have an intervention delivered (Group 2) and
3. families perceived by the health visitor *not* to require the intervention from the DHSW and therefore did not receive an intervention (Group 3).

The comparison Group 2 were families who were referred for an intervention but for various operational reasons (primarily, the DHSW was not yet in post at this early stage of implementation) the intervention was not delivered.

### Data linkage

The data linkage for this study was conducted by the ‘electronic Data Research and Innovation Service’ (eDRIS) within Information Services Division (ISD) Scotland. Probability matching techniques based on Howard Newcombe principles [8] were used to link three national health and administrative databases. Individual-level data from the following sources were linked:

- Child Health Surveillance (CHS): assessment conducted by health visitors on all newborn babies at 6–8 weeks of age between 1st September 2010 and 31st December 2012 where the referral pathway was (at least partially) operational. This dataset provided information on whether the family was referred to a DHSW for additional support or not. In addition, information on breast/bottle feeding, smoking in the household, area-based deprivation (Scottish Index of Multiple Deprivation), and an indication of the level of external support required by the family was also available.
- Health Informatics Centre (HIC): data on DHSW delivery of the intervention with families between 1st September 2010 and 31st July 2013. This dataset provided information on dates of DHSW contact with family and types of support delivered.
- Management & Dental Accounting System (MIDAS): data on child dental practice attendance between 1st September 2010 and 30th September 2013.

The endpoints for HIC and MIDAS were lagged to give sufficient time for a family to receive an intervention and be seen in dental practice.

### Statistical analyses

All analyses were undertaken using Stata (StataCorp. 2015. *Stata Statistical Software: Release 14*. College Station, TX: StataCorp LP) through a secure research portal provided by the National Safe Haven. Access was via a remote desktop through a virtual private network (VPN). A process of quality assurance was undertaken to check and clean the datasets.

Survival analysis was used to take account of unequal lengths of time spent within the cohort in which to reach the primary outcome of dental attendance. In this analysis, “time to event” represented the time taken for a child to first attend a dental practice. This was calculated from the date of birth to the date of dental practice attendance.

Life tables were produced to ascertain the median number of months taken for children to first attend a dental practive across all groups combined, and within each of the groups. Cox’s regressions were performed to model the independent effects of the intervention on attendance rates and time to first attendance in each of the three groups. The results are presented as Kaplan Meier survival curves and unadjusted and adjusted Hazard Ratios (95% confidence intervals). The proportional hazards assumption was deemed to hold by examining the plots produced for the Cox regression.

For the secondary aim, the following variables were used:

- Scottish Index of Multiple Deprivation (SIMD) (2012): this is based on the home postcode of the family. Provides a relative ranking of deprivation across Scotland which is based on seven deprivation indicators: income, employment, health, education, access to services, housing and crime. Ranks are grouped into quintiles ranging from 1 (20% most deprived) to 5 (20% least deprived).
- Risk score: this is an aggregated risk score based on area-based deprivation (SIMD), type of feeding, smoking in household, and additional support needed. Each child in the dataset was given a score of 1 for each of these four risk factors:
  - Living in the most deprived areas (SIMD 1)
  - Being bottle-fed
  - Living in a smoking household
  - Being assigned an ‘intensive’ health plan by a health visitor at 6–8 weeks

We report outcomes in relation to ‘0 risk factors’, ‘1 risk factor’ , '2 risk factors’ and ‘high risk’. ‘High risk’ is 3 or more risk factors.

## Results

### Characteristics of the sample

Across Scotland, at this early stage of rollout, the referral pathway was operational for 35236 children in total (31% of the total population assessed by health visitors (*n* = 114097)).

Health visitors referred 33% (11495) of the children they assessed to the DHSW intervention. Just under half (44% (5087)) of these children received an intervention from a DHSW (Group 1) and 56% (6408) did not, despite being referred (Group 2). The remaining children (*n* = 23741) were not considered by the health visitor to require additional support from a DHSW, and therefore did not receive an intervention (Group 3). Table 1 shows the sex, minimum, maximum, Q1 and Q3 for  age at dental practice attendance and area-based deprivation of the cohort.

**Table 1:** Sex, age at dental practice attendance and area-based deprivation of the cohort

Total	Group 1 HV: YES; DHSW: YES *N* = 5087	Group 2 HV: YES; DHSW: NO *N* = 6408	Group 3 HV: NO; DHSW: NO *N* = 23741
Sex % (n)			
Female	49.8 (2531)	47.3 (3033)	48.5 (11514)
Male	50.2 (2556)	52.7 (3375)	51.5 (12227)
Age (months)			
Minimum	1.5	1.5	1.5
Maximum	39.5	38.4	38.9
Q1	5.6	9.2	10.0
Q3	17.8	19.1	18.7
Area-based deprivation quintile (SIMD) % (n)			
Quintile 1-most deprived 20%	39.5 (2008)	32.4 (2069)	18.9 (4468)
Quintile 2	23.9 (1214)	21.5 (1377)	21.9 (5185)
Quintile 3	17.5 (887)	17.5 (1117)	21.9 (5183)
Quintile 4	11.9 (604)	14.9 (955)	22.0 (5213)
Quintile 5- least deprived 20%	7.2 (367)	13.7 (875)	15.3 (3620)

GROUP 1 are those whom the health visitor referred for DHSW intervention and received the intervention. GROUP 2 are those whom the health visitor referred for DHSW intervention but did not receive the intervention. GROUP 3 are those whom the health visitor perceived to not need a DHSW intervention and so they were neither referred nor received the intervention

### Effect of the DHSW intervention on dental attendance

The cumulative survival probabilities and median times to first attendance (survival) for the three groups are shown in Table 2.

**Table 2:** Cumulative survival probabilities, Hazard Ratios and median time to dental practice attendance for families assessed by health visitors

	Cumulative survival probabilities	Hazard ratios [95% CI]		Median time to participation [95% CI]	
Group 1 HV: YES; DHSW: YES *N = 5087*	0.88	1.87	[1.8 to 1.9]	8.8	[8.5 to 9.1]
Group 2 HV: YES; DHSW: NO *N = 6408*	0.82	1 (ref)		17.8	[17.1 to 18.6]
Group 3 HV: NO; DHSW: NO *N = 23,741*	0.81	0.98	[0.9 to 1.0]	18.4	[18.1 to 18.7]

GROUP 1 are those whom the health visitor referred for DHSW intervention and received the intervention. GROUP 2 are those whom the health visitor referred for DHSW intervention but did not receive the intervention. GROUP 3 are those whom the health visitor perceived to not need a DHSW intervention and so they were neither referred nor received the intervention

Rates of dental attendance for those who were referred to a DHSW by the health visitor and received an intervention (Group 1) were 88%, compared to 82% for those who were referred but did not receive an intervention (Group 2) and 81% for those families who were perceived to not require additional support (Group 3).

**Fig. 1:**
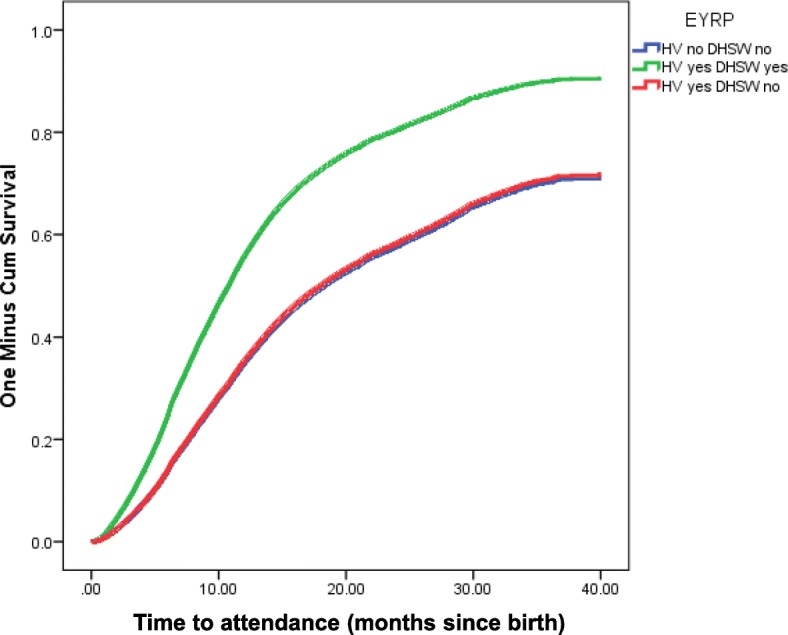
Survival curve for time to attendance at a dental practice

Children in Group 1 were more likely to attend a dental practice (HR [95% CI] =1.87 [1.8 to 1.9]) and first attended a dental practice 9 months earlier (median time until first attendance: 8.8 months versus 17.8 months) compared to Group 2. In Group 3 vs Group 2: HR [95%CI] = 0.98 [0.9 to 1.0]. The median time to first attendance for Group 3 was 18.4 months. Figure 1 presents the Kaplan Meier survival curves for time to attendance at a dental practice for each of the three groups.

### Reach of the DHSW intervention

Table 3 presents the distribution of area-based deprivation quintiles (SIMD) and individual risk scores for the whole cohort (*n* = 35236) and in all those within the cohort who were referred for DHSW support (*n* = 11495). SIMD quintiles and risk scores are also displayed according to whether an intervention was delivered (Group 1) or not (Group 2).

**Table 3:** Distribution of area-based deprivation quintiles and individual risk scores in all children referred for DHSW intervention and, separately, in those referred who received an intervention (Group 1) and who did not (Group 2)

	Group 1	Group 2	Referred Group	Total cohort
*N*	5087	6408	11495	35236
SIMD % (*n*)				
Q1-most deprived	39.5 (2008)	32.3 (2069)	35 (4077)	24.4 (8525)
Q2	23.9 (1214)	21.5 (1377)	23 (2591)	22.2 (7776)
Q3	17.4 (887)	17.4 (1117)	17.4 (2004)	20.6 (7187)
Q4	11.9 (604)	14.9 (955)	13.6 (1559)	19.2 (6772)
Q5-least deprived	7.2 (367)	13.7 (875)	10.8 (1242)	13.6 (4862)
*missing	0.001 (7)	0.002 (15)	0.002 (22)	*N* = 114
Risk score % (*n*)				
0- low risk	48.3 (2461)	52.7 (3375)	50.8 (5836)	60.4 (21248)
1	39.7 (2022)	36.8 (2355)	38.1 (4377)	32.2 (11346)
2	10.8 (549)	9.7 (621)	10.2 (1170)	6.9 (2431)
3 or more- high risk	1.0 (55)	0.9 (57)	0.9 (112)	0.6 (211)

GROUP 1 are those whom the health visitor referred for DHSW intervention and received the intervention. GROUP 2 are those whom the health visitor referred for DHSW intervention but did not receive the intervention. REFERRED GROUP are all those referred for a DHSW intervention by a health visitor. TOTAL COHORT is all those referred and not referred

Health visitors referred more families from deprived areas to the intervention (35% in SIMD Q1 Referred Group vs 24.4% in Total Cohort), and a slightly higher percentage of children with greater (‘2’ or ‘3 or more’) risk scores (11.1% vs 7.5% in total cohort), in line with the targeted nature of the intervention.

There is little difference in the distribution of SIMD or risk score between Groups 1 and 2, with no indication that easier to reach families were being selected by the DHSW to reduce the risk of the intervention failing.

## Discussion

Using a quasi-experimental approach, this study has shown that community-based link workers within a national oral health improvement programme (Childmsile) are effective at linking targeted families of young children to a dental practice *and* at an earlier age for the delivery of ongoing prevention.

There is some targeting of the DHSW intervention towards families living in more deprived areas, and those experiencing multiple risk factors that may indicate they require additional support, as the programme intended.

To our knowledge, this is the first time that a quasi-experimental approach has been taken to evaluate community-based link workers on a population wide scale, using linkage of large health and administrative datasets (unique to Scotland). Quasi-experimental designs are becoming more commonly used to evaluate large public health interventions that are not amenable to experimental manipulation and that rely on linkage of secondary datasets [9]. As a result, time, resource and logistical issues associated with experimental studies, (such as screening, randomization, primary data collection) are minimised. The large sample size provided robust estimates of effectiveness of the intervention and allowed assessment of subgroups. We were able to test the effectiveness of the DHSW intervention due to the natural rollout of Childsmile, which meant that some targeted families did not receive the intervention, due to operational issues rather than non-compliance with the intervention, thereby improving the internal validity of the study.

This study was undertaken as part of a wider theory-based evaluation of the Childsmile programme [10, 11]. This ensured that study outcomes, specified a priori, were in keeping with agreed programme theory explicating the DHSW role, and measured the primary expected outcome of the DHSW intervention [12].

In all non-randomized studies, lack of randomization into exposure groups can limit generalizability and the ability to infer causality. Confounding in quasi-experimental studies often arises; however, we did not observe differences between exposure groups at baseline of key confounders (area-based deprivation quintiles or individualised risk scores). We cannot however, rule out the possibility of residual confounding or the potential that there are unmeasured confounders.

Due to the nature of this study, it was not possible to evaluate the content or quality of the interventions delivered by the DHSWs or how variable this delivery was across the country, and how this may change over time as the programme matures. Qualitative data on the impact of DHSW support from the service-user perspective is also, as yet, unavailable. However, in-depth qualitative studies are currently ongoing to address some of these questions.

This study’s results are in-keeping with recent (weak) evidence suggesting that community-based link workers can be successful at linking families with community resources and, in doing so, may impact positively on health and health behaviours [2–4]. More specifically, previous evaluations of oral health improvement interventions in Scotland have shown associations between the use of link workers and increasing levels of dental registration and improving oral health outcomes. A cross-sectional analysis of the Starting Well programme found that more mothers receiving the ‘Starting Well’ intervention reported that they had registered their infant at a dental practice than those receiving the generic service (OR = 2.74, *p* < 0.001) [13]. Similarly, a programme trialled the use of volunteer community activists to deliver oral health promotion in the most deprived areas of Glasgow. A significant increase in the percentage of children with no obvious dental decay was found in areas where the intervention was implemented [14]. Although indicative of a beneficial effect of community link workers on oral health, limitations in the design of these studies, has meant causal interpretations have not been possible.

Additionally, the literature continues to lack consensus on which aspects of community link worker roles are the ‘active ingredients’ in bringing about engagement with services, and what other roles/duties might enhance the outcomes for these families.

Having demonstrated the effectiveness of the DHSW in facilitating participation at dental practice, we recognise that this is an interim outcome for the Childsmile programme and what is ultimately important is the effect this increased participation has on the long-term outcomes of improved oral health and a reduction in inequalities. In addition, it will be important to assess the content and quality (e.g. the type and frequency of support offered to families based on particular characteristics/needs) of the delivery of the DHSW intervention to establish the degree to which the intervention is being tailored to individual needs and the extent to which variation in delivery influences outcomes. This work is part of the wider evaluation of the Childsmile programme.

## Conclusions

This study has shown that link workers (DHSWs) are effective at linking disadvantaged children to primary care dental services and, most notably, *at a younger age* for delivery of preventative treatment. This is the first study of its kind to evaluate the effectiveness of link-worker programmes using a robust quasi-experimental design on a large linked dataset. These results will inform future health improvement programmes which aim to reach and support families from more disadvantaged backgrounds to improve health and reduce inequalities.

## Abbreviations

DHSW: Dental Health Support Worker
EYRP: Early Years Referral Pathway
HR: Hazard Ratio
NHS: National Health Service
OR: Odds Ratio
SIMD: Scottish Index of Multiple Deprivation
